# Molecular Epidemiology and Colistin-Resistant Mechanism of *mcr*-Positive and *mc*r-Negative *Escherichia coli* Isolated From Animal in Sichuan Province, China

**DOI:** 10.3389/fmicb.2022.818548

**Published:** 2022-03-29

**Authors:** Fulei Li, Ping Cheng, Xiaoting Li, Ruimeng Liu, Haibin Liu, Xiuying Zhang

**Affiliations:** ^1^Heilongjiang Key Laboratory for Animal Disease Control and Pharmaceutical Development, Northeast Agricultural University, Harbin, China; ^2^Department of Basic Veterinary Science, College of Veterinary Medicine, Northeast Agricultural University, Harbin, China

**Keywords:** colistin resistant mechanism, *mcr*, *Escherichia coli*, multidrug resistant, two-component systems

## Abstract

Colistin is the last line of defense for the treatment of multidrug-resistant gram-negative bacterial infections. However, colistin resistance is gradually increasing worldwide, with resistance commonly regulated by two-component system and *mcr* gene. Thus, this study aimed to investigate molecular epidemiology and colistin-resistant mechanism of *mcr*-positive and *mcr*-negative *Escherichia coli* isolates from animal in Sichuan Province, China. In this study, a total of 101 colistin-resistant *E. coli* strains were isolated from 300 fecal samples in six farms in Sichuan Province. PCR was used to detect *mcr* gene (*mcr-1* to *mcr-9*). The prevalence of *mcr-1* in colistin-resistant *E. coli* was 53.47% (54/101), and the prevalence of *mcr-3* in colistin-resistant *E. coli* was 10.89% (11/101). The colistin-resistant *E. coli* and *mcr-1*–positive *E. coli* showed extensive antimicrobial resistance profiles. For follow-up experiments, we used 30 *mcr*-negative and 30 *mcr-1*–positive colistin-resistant *E. coli* isolates and *E. coli* K-12 MG1655 model strain. Multi-locus sequence typing (MLST) of 30 strains carrying *mcr-1* as detected by PCR identified revealed six strains (20%) of ST10 and three strains (10%) of each ST206, ST48, and ST155 and either two (for ST542 and 2539) or just one for all other types. The conjugation experiment and plasmid replicon type analysis suggest that *mcr-1* was more likely to be horizontally transferred and primarily localized on IncX4-type and IncI2-type plasmid. The ST diversity of the *mcr-1* indicated a scattered and non-clonal spreading in *mcr-1*–positive *E. coli.* Twenty-eight *mcr*-negative colistin-resistant *E. coli* isolates carried diverse amino acid alterations in PmrA, PmrB, PhoP, PhoQ, and MgrB, whereas no mutation was found in the remaining isolates. The finding showed the high prevalence of colistin resistance in livestock farm environments in Sichuan Province, China. Our study demonstrates that colistin resistance is related to chromosomal point mutations including the two-component systems PhoP/PhoQ, PmrA/PmrB, and their regulators MgrB. These point mutations may confer colistin resistance in *mcr*-negative *E. coli*. These findings help in gaining insight of chromosomal-encoded colistin resistance in *E. coli*.

## Introduction

Colistin is the last line of defense for the treatment of multidrug-resistant (MDR) gram-negative bacteria (Paterson et al., 2020). The rate of colistin resistance has risen to 30% of Carbapenem-resistant Enterobacteriaceae (CRE) isolates in Italy, Spain, and Greece over the past decade (Stefaniuk and Tyski, 2019). Colistin resistance is a growing public health concern worldwide. Colistin is a cyclic polycationic peptide that interacts with anionic lipopolysaccharide (LPS) molecules (Olaitan et al., 2014). However, overuse and misuse of colistin accelerate propagation of antibiotic resistance genes. The exact mechanism of colistin resistance is not well understood. It has been shown that an altered outer membrane, a loss of lipid A, and increase in drug efflux pumps are associated with colistin resistance (Raetz et al., 2007; Moffatt et al., 2010; Padilla et al., 2010).

The plasmid-mediated colistin resistance was not demonstrated until 2015. Liu et al. (2016) first systematically reported *mcr-1* gene. Over the past few years, *mcr-1* became common all over the world. The *mcr-1* has the potential to spread rapidly by horizontal transfer and may pose a significant public health risk (Rapoport et al., 2016; Rolain et al., 2016; Sonnevend et al., 2016; Borowiak et al., 2017). In the past few years, novel plasmid-encoded colistin resistance genes have also been identified. So far, novel colistin resistance genes (*mcr-2* to *mcr-10*) have been reported (Xavier et al., 2016; Carattoli et al., 2017; Yin et al., 2017; AbuOun et al., 2018; Yang et al., 2018; Wang et al., 2019, 2020). The *mcr-2* to *mcr-9* genes encode the proteins MCR-2 to MCR-9, which share 81, 32.5, 34, 36, 83, 35, 31, and 36% amino acid sequence identity with MCR-1, respectively (Carroll et al., 2019; Nang et al., 2019). MCR-10 is encoded by *mcr-10*, which contains 82.93% amino acids identical to that in MCR-9 (Wang et al., 2020). The *mcr-1* has spread to more than 60 countries (Cheng et al., 2021). It has been reported that prevalence of *mcr-1*–positive bacteria ranged from 0.35 to 36.00% in pigs and 2.40–30.00% in poultry (Xiaomin et al., 2020). To date, *mcr-1* was identified in many different plasmid types, including IncI2, IncHI2, IncX4, IncP, IncY, and IncF (Shen et al., 2018).

Before the discovery of *mcr* gene, clinical colistin resistance was associated with mutation of chromosomal gene (Kim et al., 2019; Mendes Oliveira et al., 2019). Chromosome-mediated colistin resistance had been linked to LPS modifications, which was associated with PmrAB or PhoPQ two-component systems (Poirel et al., 2017). MgrB is a negative regulator of the PhoPQ system. The inactivation of MgrB leads to over expression of the phoPQ operon (Cheng et al., 2010; Cannatelli et al., 2013). Moreover, amino acid substitutions of PmrA and/or PmrB have been reported in clinical isolates of *Acinetobacter baumanni* and *Klebsiella pneumoniae* (Poirel et al., 2017). First, amino acid substitutions may affect protein function. In addition, amino acid substitutions in MgrB, PmrA/B, and PhoP/Q are a common mechanism of colistin resistance among *K. pneumoniae* in clinical settings (Luo et al., 2017). However, amino acid substitutions in MgrB, PmrA/B, and PhoP/Q are rarely reported among *mcr*-negative colistin-resistant *Escherichia coli* isolates. A recent study showed that missense mutations may be responsible for colistin resistance (Olaitan et al., 2014).

This study aimed to investigate molecular epidemiology and colistin-resistant mechanism of *mcr*-positive and *mcr*-negative *E. coli* isolated from animals in Sichuan Province, China.

## Materials and Methods

### Sample Collection

From January 2016 to March 2018, a total of 300 fecal swabs were collected from six different farms in Sichuan Province. The samples were kept on ice and were immediately brought to laboratory. The samples were cultured on MacConkey agar at 37°C for 18–24 h, and then, five colonies with typical *E. coli* morphology were selected. The *E. coli* were identified using biochemical methods and confirmed by PCR amplification of *16S rRNA* and sequencing. The primer of *16S rRNA* is listed in Supplementary Table 1. The protocols used during this study were approved by the Northeast Agricultural University Institutional Animal Care and Use Committee, and all the animal care and treatment methods complied with the standards described in the Laboratory Animal Management Regulations (revised 2016) of Heilongjiang Province, China.

### Detection of Colistin-Resistant *Escherichia coli* Isolates and *mcr* Gene

To screen colistin-resistant *E. coli*, *E. coli* were cultured on MacConkey agar at 37°C for 18–24 h. To determine the colistin minimum inhibitory concentration (MIC), *E. coli* isolates (>4μg/ml) were served as colistin-resistant *E. coli*. A DNA extraction kit (TIANGEN, Beijing, China) was used to extract genomic DNA of colistin-resistant *E. coli* isolates. The *mcr*-harboring isolates were screened by PCR amplification and were validated by sequencing. The primers of *mcr* gene are listed in Supplementary Table 1.

### Antimicrobial Susceptibility Testing

The susceptibility of colistin-resistance *E. coli* isolates to 23 antibiotics, namely, Nitrofurantoin, Ciprofloxacin, Levofloxacin, Kanamycin, Amikacin, Amoxicillin, Tigecycline, Cefepime, Chloramphenicol, Fosfomycin, Aztreonam, Ampicillin, Ampicillin/sulbactam, Cefoxitin, Doxycycline, Streptomycin, Ceftriaxone, Florfenicol, Cefuroxime, Sulfamethoxazole, Gentamicin, and Tetracycline, was determined by the standard disk diffusion method in accordance with the Clinical and Laboratory Standards Institute (CLSI).^1^ *Escherichia coli* ATCC 25922 was served as a quality control strain for susceptibility testing.

### Detection of Antimicrobial Resistance Genes

The presence of the β-lactamase genes (*bla_*CTX*–*M*_*, *bla*_*TEM*_, and *bla*_*SHV*_) (Dallenne et al., 2010), aminoglycoside resistance genes (*strA*, *strB*, *aacC2*, and *aacC4*) (Kozak et al., 2009), tetracycline resistance genes (*tetA*, *tetB*, and *tetC*) (Ji et al., 2020), fluoroquinolone resistance genes [*qnrS*, *oqxA*, *oqxB*, *qepA*, and *aac(6′)-Ib-cr*] (Ciesielczuk et al., 2013), florfenicol resistance gene (*floR*) (Lu et al., 2018), and sulfonamide resistance genes (*sul1*, *sul2*, and *sul3*) (Hammerum et al., 2006) were examined by PCR amplification and were validated by sequencing. The primers of resistance genes are listed in Supplementary Table 2. The obtained DNA sequences were analyzed using ChromasPro software and were compared with published sequences by BLAST.

### Detection of *pmrA*/*B*, *mgrB*, and *PhoP*/*Q* Amino Acid Variants

The entire *pmrAB*, *mgrB*, and *phoPQ* genes were amplified of colistin-resistant isolates using the primers listed in Supplementary Table 3. The amplification products were validated with Sanger sequencing. Amino acid sequences of *mcr*-negative colistin-resistant *E. coli* isolates were compared with the reference strain *E. coli* K-12 MG1655. Missense mutations of PmrA/B, PhoP/Q, and MgrB identified in *mcr*-negative colistin-resistant *E. coli* were analyzed using the PROVEAN (Choi and Chan, 2015) prediction software. In addition, amino acid substitutions were considered “deleterious” if the PROVEAN score was ≤-2.5 and “neutral replacements” if the PROVEAN score was >-2.5 (Choo et al., 2016; Higuchi et al., 2016). We used PROVEAN bioinformatic tool^2^ to predict whether amino acid substitutions in MgrB, PmrA/B, and PhoP/Q affect protein function (Choi et al., 2012). SMART (Simple Modular Architecture Research Tool) is a web resource,^3^ providing simple identification and extensive annotation of protein domains and the exploration of protein domain architectures (Schultz et al., 1998; Letunic et al., 2015). SMART analysis was performed to determine the domain architectures of PmrA, PmrB, PhoP, PhoQ, and MgrB protein.

### Conjugation Experiment and Plasmid Replicon Type Analysis

The transferability of *mcr-1* was tested by conjugation experiment with 30 *mcr-1*–positive *E. coli* (MCRPEC) as donors and *E. coli* J53 as recipient strains. MacConkey agar plates containing rifampicin (256 μg/ml) and colistin (4 μg/ml) were used to select *mcr-1*–positive transconjugants. The *mcr-1* gene of transconjugants was examined by PCR amplification and was validated by sequencing. The replicon types of the transconjugants were determined according to previous studies (Carattoli et al., 2005).

### Multi-Locus Sequence Typing Analysis

Sequence type of 30 MCRPEC was determined according to the primers and protocol specified in *E. coli* multi-locus sequence typing (MLST) database website, which is based on the housekeeping genes *adk*, *fumC*, *gyrB*, *icd*, *mdh*, *purA*, and *recA*. The primers of housekeeping gene are listed in Supplementary Table 4. The obtained DNA sequence alignments were performed by using ChromasPro software. The phylogenetic tree was constructed by the neighbor-joining method.

## Results and Discussion

### Prevalence of *mcr*-Positive *Escherichia coli* Isolated From Animal

**FIGURE 1:**
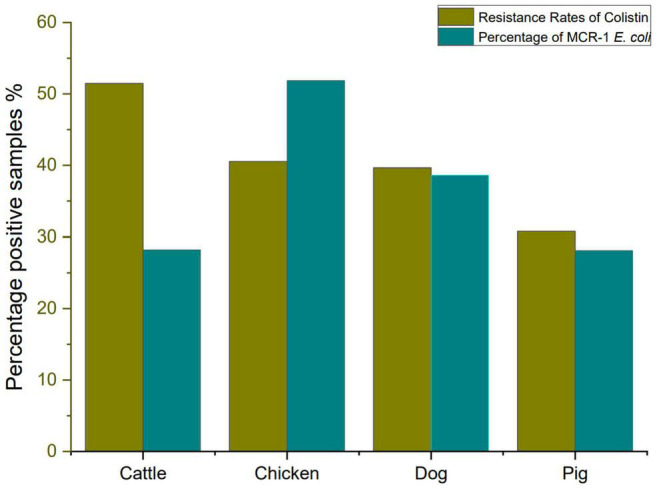
The resistance rate to colistin and percentage of *mcr-1*–positive *Escherichia coli.*

In this study, we investigate colistin resistance rate in *E. coli* isolated from six different farms in Sichuan Province during 2016–2018. A total of 254 *E. coli* strains were isolated from 300 fecal samples, including 37 chickens, 91 pigs, 68 cattle, and 58 dogs. The *E. coli* isolates from cattle showed a high resistance rate to colistin (51.47%, 35/68), followed by *E. coli* isolates from chicken (40.54%, 15/37), *E. coli* isolates from dog (39.66%, 23/58), and *E. coli* isolates from pig (30.77%, 28/91). However, it has been reported that the prevalence of colistin resistance in *E. coli* isolated from farms in different areas of China during 2013–2014, which revealed that colistin resistance rates in *E. coli* from pigs, chickens, and cattle were 26.5, 14.0, and 0.9%, respectively (Zhuge et al., 2019). Our data also showed that colistin resistance rate has risen significantly high. Colistin-resistant *E. coli* isolates (MIC of colistin ≥ 4 μg/ml) are listed in Supplementary Table 5. The resistance rate to colistin and percentage of MCRPEC are shown in Figure 1.

However, discoveries of plasmid-mediated colistin resistance gene in many countries have heralded a significant threat to public health worldwide (Kai and Wang, 2020). As shown in Table 1, *mcr-1* gene was detected in 54 (53.47%) out of the selected 101 isolates, and *mcr-3* gene was detected in 11 (10.89%) out of the selected 101 isolates. Eleven *E. coli* isolates have both *mcr-1* and *mcr-3* gene. No other *mcr* genes were found in this survey. Similar to our result, a surveillance of colistin resistance performed in Jiangsu Province revealed that the *mcr-1* prevalence was 68.86% in pigs (Zhang et al., 2019). More detailed studies of *mcr-1* have been performed in colistin-resistant isolates (Tong et al., 2018). The previous study showed that *mcr-1* had been identified in approximately 60 countries across five different continents (Cao et al., 2018; Sun et al., 2018; Wang et al., 2018). The finding of mobilizable *mcr*-like genes became a global concern due to the possibility of horizontal transfer of the plasmid that often carry resistance determinants to beta-lactams and/or quinolones (Mendes Oliveira et al., 2019).

**TABLE 1:** Number of *mcr*-positive and *mcr*-negative colistin-resistant *Escherichia coli* in this study.

Origin	Period of isolation	No. *E. coli*	No. of *mcr*-positive			No. *mcr*-negative colistin-resistant *E. coli*
			*E. coli*			
			
			*mcr-1*	*mcr-3*	*mcr-1/3*	
Chicken	2016–2018	37	12	–	–	4
Pig	2016–2018	91	16	–	–	12
Cattle	2016–2018	68	12	4	4	23
Dog	2016–2018	58	14	7	7	9

*mcr-1/3 strains have both mcr-1 and mcr-3 gene.*

### Antimicrobial Susceptibility of Colistin-Resistance and *mcr-1*–Positive *Escherichia coli*

**FIGURE 2:**
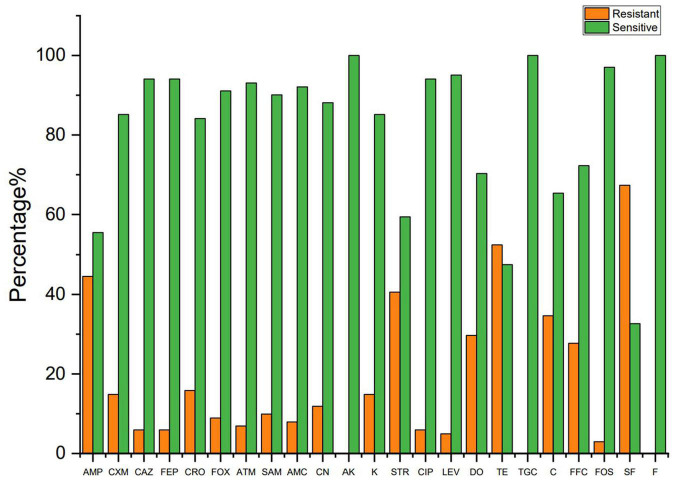
The resistant rate of colistin resistance *E. coli* to other antibiotics. AMP, ampicillin; CXM, cefuroxime; CAZ, ceftazidime; FEP, cefepime; CRO, ceftriaxone; FOX, cefoxitin; ATM, aztreonam; SAM, ampicillin-sulbactam; AMC, amoxicillin–clavulanic acid; CN, gentamicin; AK, amikacin; K, kanamycin; STR, streptomycin; CIP, ciprofloxacin; LEV, levofloxacin; TE, tetracycline; DO, doxycycline; TGC, tigecycline; C, chloramphenicol; FFC, florfenicol; FOS, fosfomycin; SF, sulfisoxazole; F, nitrofurantoin.

The susceptibility of 101 colistin-resistant *E. coli* isolates to 23 antimicrobials was determined by the standard disk diffusion method. Figure 2 shows a significant difference in resistance rate of the *E. coli* isolates to 23 antibiotics. Among the colistin-resistant *E. coli* isolates, all isolates were susceptible to amikacin, tigecycline, and nitrofurantoin. Antibiotic sensitivity tests revealed that colistin-resistant *E. coli* isolates have a highly resistance to sulfisoxazole; a moderate rate of resistance to ampicillin, streptomycin, and tetracycline; and a low rate of resistance to cefuroxime, ceftazidime, cefepime, ceftriaxone, cefoxitin, aztreonam, ampicillin-sulbactam, amoxicillin-clavulanic acid, gentamicin, kanamycin, ciprofloxacin, and levofloxacin. The colistin-resistant *E. coli* isolates displayed high resistance rates to antibiotics that are commonly used in veterinary medicine in Sichuan Province (Ma et al., 2017; Zhang et al., 2021), including sulfisoxazole, ampicillin, streptomycin, tetracycline, and chloramphenicol. The antimicrobial resistance rates have reached to 30%, which may be due to the breadth of our sample or because of the generally high use of antibiotics in Sichuan Province. Three studies reported the rate of resistance to streptomycin was within the range 0–7% (Routman et al., 1985; Sayah et al., 2005; Tong et al., 2018). However, the rate of resistance to streptomycin has reached 30% in this study. This difference may reflect difference in antimicrobial use in different livestock.

**FIGURE 3:**
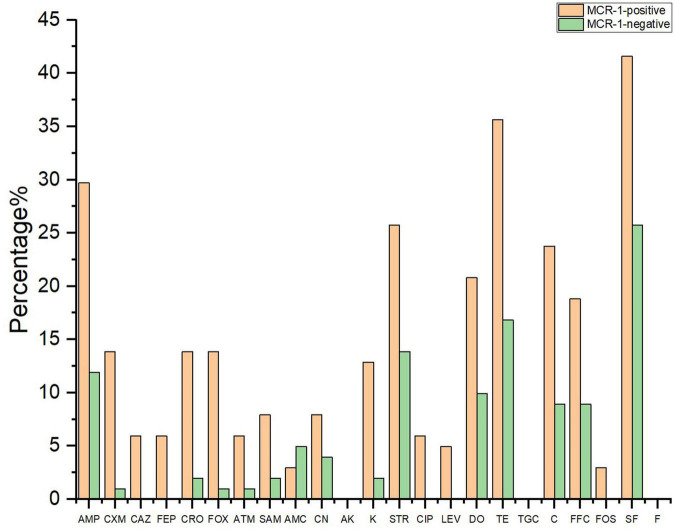
The resistant rate of *mcr-1*–positive and *mcr-1*–negative *E. coli* to 23 antibiotics. AMP, ampicillin; CXM, cefuroxime; CAZ, ceftazidime; FEP, cefepime; CRO, ceftriaxone; FOX, cefoxitin; ATM, aztreonam; SAM, ampicillin–sulbactam; AMC, amoxicillin–clavulanic acid; CN, gentamicin; AK, amikacin; K, kanamycin; STR, streptomycin; CIP, ciprofloxacin; LEV, levofloxacin; TE, tetracycline; DO, doxycycline; TGC, tigecycline; C, chloramphenicol; FFC, florfenicol; FOS, fosfomycin; SF, sulfisoxazole; F, nitrofurantoin.

Figure 3 shows the resistance rate of the MCRPEC isolates to 23 antibiotics. MCRPEC isolate have a moderate rate of resistance (20–60%) to ampicillin, streptomycin, doxycycline, tetracycline, and sulfisoxazole; and a low rate of resistance (<20%) to cefuroxime, ceftazidime, cefepime, ceftriaxone, cefoxitin, aztreonam, ampicillin-sulbactam, amoxicillin-clavulanic acid, gentamicin, kanamycin, ciprofloxacin, levofloxacin, and fosfomycin. All the MCRPEC isolate were susceptible to amikacin, tigecycline, and nitrofurantoin. The resistance phenotype of *mcr-1* positive *E. coli* is shown in Supplementary Table 6.

### Detection of Antimicrobial Resistance Genes of *mcr-1*–Positive *Escherichia coli*

**FIGURE 4:**
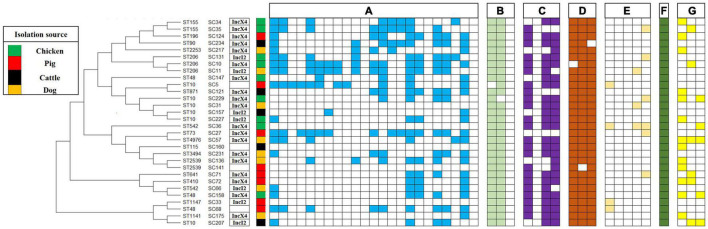
Phylogeny of *mcr-1*–positive *E. coli* (sequence types, strain number, plasmid type, source of isolation, and map of phenotypic resistance and resistance genes). Full and empty square mean presence and absence of antimicrobial resistance (AMR) gene, respectively, whereas empty space means non-defined. **(A)** Resistance profiles: ampicillin, cefuroxime, ceftazidime, cefepime, ceftriaxone, cefoxitin, aztreonam, ampicillin-sulbactam, amoxicillin-clavulanic acid, gentamicin, amikacin, kanamycin, streptomycin, ciprofloxacin, levofloxacin, tetracycline, doxycycline, tigecycline, chloramphenicol, florfenicol, fosfomycin, sulfisoxazole, and nitrofurantoin. **(B)** Beta-lactam resistance genes: *bla_*CTX*–*M*_, bla_*TEM*_* and *bla*_*SHV*_. **(C)** Aminoglycoside resistance genes: *strA*, *strB*, *aacC2*, and *aacC4*. **(D)** Tetracycline resistance genes: *tetA*, *tetB*, and *tetC*. **(E)** Fluoroquinolone resistance genes: *qnrS*, *oqxA*, *oqxB*, *qepA*, and *aac(6′)-Ib-cr*. **(F)** Florfenicol resistance gene: *floR*. **(G)** Sulfonamide resistance genes: *sul1*, *sul2*, and *sul3*.

The prevalence of additional antimicrobial resistance genes in 30 MCRPEC is shown in Figure 4. Overall, *bla*_*TEM*_ (*n* = 29, 96.67%) and *bla*_*CTX–M*_ (*n* = 29, 96.67%) were the most common ESBL genes, and *bla*_*SHV*_ was not detected in this study. The aminoglycoside resistance genes with the highest detection rate were *aacC2* (*n* = 24, 80.00%) and *aacC4* (*n* = 25, 83.33%), followed by *strA* (*n* = 23, 76.67%), and *strB* were not detected. Among tetracycline resistance genes, *tetA* (*n* = 29, 96.67%), *tetB* (*n* = 29, 96.67%), and *tetC* (*n* = 29, 96.67%) were the most common tetracycline resistance genes. The fluoroquinolone resistance genes with the highest detection rate were *aac(6′)-Ib-cr* (*n* = 6, 20.00%), followed by *qepA* (*n* = 4, 13.33%) and *qnrS* (*n* = 4, 13.33%), and *oqxA* and *oqxB* were not detected in our study. The florfenicol resistance gene *floR* detection rate was (*n* = 30, 100.00%). The sulfonamide resistance genes with the highest detection rate were *sul1* (*n* = 13, 43.33%), followed by *sul2* (*n* = 7, 23.33%) and *sul3* (*n* = 6, 20.00%). In our study, a high prevalence of ESBL genes (*bla*_*TEM*_ and *bla*_*CTX–M*_), aminoglycoside resistance genes (*aacC2*, *aacC4*, and *strA*), tetracycline resistance genes (*tetA*, *tetB*, and *tetC*), and florfenicol resistance gene *floR* were found in 30 MCRPEC. This result may reflect that plasmid harboring *mcr-1* usually carry other resistance genes (Rozwandowicz et al., 2018; Cheng et al., 2021).

### Transferability and Plasmid Replicon Types of *mcr-1*–Positive *Escherichia coli*

As shown in Figure 4, the conjugation result showed that 27 isolates successfully conjugated among 30 representative MCRPEC. The success rate of conjugation was as high as 90%. *mcr-1* has been observed in two types plasmids, including IncX4 (*n* = 19) and IncI2 (*n* = 7). In this study, the transferability of MCRPEC was assessed among 30 representative strains. The result was in line with previous study, which showed that majority of *mcr-1*–carrying plasmids were transferable (Cheng et al., 2021). Previous research has shown *mcr-1*–carrying plasmids belong to different replicon types, including IncI2 (Matamoros et al., 2017), IncHI1 (Zurfluh et al., 2016), IncHI2 (Matamoros et al., 2017), IncFIB (Wang et al., 2017; Khezri et al., 2020), IncFII (Xavier et al., 2016), IncP (Zhao et al., 2017), IncX4 (Gao et al., 2016), and IncY (Shen et al., 2018).

### Molecular Genotyping of *mcr-1*–Positive *Escherichia coli*

As shown in Figure 4, 30 MCRPEC isolates were assigned to 17 STs. ST10 (*n* = 6) was the most numerous ST in this study, followed by ST206 (*n* = 3), ST48 (*n* = 3), ST155 (*n* = 3), ST542 (*n* = 2), and ST2539 (*n* = 2), and then by single ST type isolates, including ST196, ST90, ST2253, ST871, ST73, ST4976, ST3494, ST641, ST410, ST1147, and ST1141. Hence, colistin-resistant *E. coli* isolates comprised a variety of STs and were therefore genetically different, with the nosocomial transmission excluded. The ST diversity of the *mcr-1*–harboring *E. coli* isolates indicated a scattered and non-clonal prevalence. The discovery of the superbug MCRPEC has triggered a huge amount of innovative scientific inquiry (Yuan et al., 2021). Recently, a study analyzed 616 whole genomes of MCRPEC isolates from NCBI online database. Similar to our result, ST10 was the most common ST among the *mcr-1*–positive isolates (Zhuge et al., 2019).

### Amino Acid Variations of *PmrA/B*, *PhoP*/*Q*, and *MgrB* in *mcr*-Negative Isolates

As shown before, colistin resistance was reported to be associated with chromosomal mutations. It is well-known that two-component PhoPQ and PmrAB were associated with LPS modification (Luo et al., 2017). LPS may play an important regulatory role in colistin-resistance isolates (Schurek et al., 2009; Kandehkar Ghahraman et al., 2021). To determine chromosome-mediated colistin-resistant mechanism in *mcr*-negative colistin-resistant isolates, we explored whether alterations in amino acid of PmrAB, PhoPQ, and MgrB affect protein function. We selected K-12 MG1655 *E. coli* as negative controls. The mutations are shown in Tables 2, 3, and we found that many variations were synonymous and non-synonymous mutations. The *mgrB* gene encodes a short 47–amino acid transmembrane protein. MgrB is a small transmembrane protein of 47 amino acids, which acts as negative feedback regulator of the PhoPQ two-component regulatory system (Lippa and Goulian, 2009). The multiple sequence alignment tool MEGA-X was used for multiple protein sequence alignment. Two mutations M1V (23 *mcr*-negative isolates) and V8A (one *mcr*-negative isolate) were detected. The mutation M1V [PROVEAN score = -4.670 (cutoff = -2.5)] and V8A [PROVEAN score = -2.808 (cutoff = -2.5)] were deleterious affecting protein function. We found similar mutations in MgrB (V8A) with previous studies (Delannoy et al., 2017; Luo et al., 2017). The mutation V8A may confer colistin resistance in *mcr*-negative *E. coli*.

**TABLE 2:** Mutations of PmrA in *mc*r-negative colistin-resistant *E. coli* isolates.

Isolates	PmrA										
	
	S29	T31	T79	T85	L116	R118	E126	I128	R139	G144	T151
*Escherichia coli* K12	**S**	**T**	**T**	**T**	**L**	**R**	**E**	**I**	**R**	**G**	**T**
*Escherichia coli* SC8	**G**										
*Escherichia coli* SC9	**G**			**A**				**N**	**P**	**S**	**A**
*Escherichia coli* SC10											
*Escherichia coli* SC11										**S**	
*Escherichia coli* SC12	**G**										
*Escherichia coli* SC13	**G**										
*Escherichia coli* SC14	**G**										
*Escherichia coli* SC15	**G**										
*Escherichia coli* SC16	**G**										
*Escherichia coli* SC17	**G**										
*Escherichia coli* SC18	**G**						**K**				
*Escherichia coli* SC19	**G**					**F**					
*Escherichia coli* SC20	**G**		**A**		**V**						
*Escherichia coli* SC21	**G**										
*Escherichia coli* SC22	**G**										
*Escherichia coli* SC23	**G**										
*Escherichia coli* SC24	**G**										
*Escherichia coli* SC25	**G**										
*Escherichia coli* SC26	**G**										
*Escherichia coli* SC27											
*Escherichia coli* SC28	**G**										
*Escherichia coli* SC29	**G**										
*Escherichia coli* SC30	**G**										
*Escherichia coli* SC31											
*Escherichia coli* SC32											
*Escherichia coli* SC33	**G**	**S**									
*Escherichia coli* SC34											
*Escherichia coli* SC35	**G**										
*Escherichia coli* SC36	**G**										
*Escherichia coli* SC37	**G**										

**TABLE 3:** Mutations of *PmrB*, *PhoP*, and *PhoQ* in *mc*r-negative colistin-resistant *E. coli* isolates.

Isolates	PmrB				MgrB		PhoP	PhoQ
				
	T235	D283	V351	Y358	M1	V8	A416	K46
*Escherichia coli* K12	**T**	**D**	**V**	**Y**	**M**	**V**	**A**	**K**
*Escherichia coli* SC8								
*Escherichia coli* SC9		**G**	**I**		**V**			
*Escherichia coli* SC10					**V**	**A**		
*Escherichia coli* SC11								
*Escherichia coli* SC12								
*Escherichia coli* SC13					**V**			
*Escherichia coli* SC14					**V**			
*Escherichia coli* SC15					**V**			
*Escherichia coli* SC16					**V**			
*Escherichia coli* SC17					**V**			
*Escherichia coli* SC18		**G**		**N**	**V**			
*Escherichia coli* SC19					**V**			
*Escherichia coli* SC20					**V**			
*Escherichia coli* SC21		**G**		**N**	**V**			
*Escherichia coli* SC22					**V**			
*Escherichia coli* SC23					**V**		**T**	
*Escherichia coli* SC24	**N**	**G**		**N**	**V**			
*Escherichia coli* SC25		**G**		**N**	**V**			
*Escherichia coli* SC26		**G**		**N**	**V**			
*Escherichia coli* SC27		**G**		**N**				
*Escherichia coli* SC28		**G**		**N**	**V**			**T**
*Escherichia coli* SC29		**G**		**N**	**V**			
*Escherichia coli* SC30		**G**		**N**	**V**			
*Escherichia coli* SC31		**G**		**N**				
*Escherichia coli* SC32					**V**			
*Escherichia coli* SC33								
*Escherichia coli* SC34								
*Escherichia coli* SC35					**V**			
*Escherichia coli* SC36					**V**			
*Escherichia coli* SC37					**V**			

**FIGURE 5:**
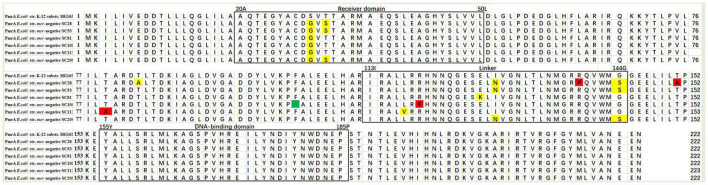
Multiple sequence alignment of PmrA across seven *mcr*-negative colistin-resistant *E. coli*. Mutations are shown with yellow boxes. The red boxes show that mutations were deleterious, affecting protein function. Amino acid deletion is shown with green box.

The *pmrA* gene encodes a short 222–amino acid transmembrane protein. The multiple sequence alignment tool MEGA-X was used for multiple protein sequence alignment. As shown in Table 2, 11 mutations S29G (24 *mcr*-negative isolates), T31S (one *mcr*-negative isolate), T79A (one *mcr*-negative isolate), T85A (one *mcr*-negative isolate), L116V (one *mcr*-negative isolate), R118F (one *mcr*-negative isolate), E126K (one *mcr*-negative isolate), I128N (one *mcr*-negative isolate), R139P (one *mcr*-negative isolate), G144S (two *mcr*-negative isolates), and T151A (one *mcr*-negative isolate) were detected. The mutations T79A [PROVEAN score = -4.586 (cutoff = -2.5)], R118F [PROVEAN score = -7.740 (cutoff = -2.5)], R139P [PROVEAN score = -3.456 (cutoff = -2.5)], and T151A [PROVEAN score = -2.790 (cutoff = -2.5)] were deleterious, affecting protein function. Figure 5 shows multiple sequence alignment of PmrA across seven *mcr-1*–negative colistin-resistant isolates. Mutations are shown with yellow boxes. The red boxes show that mutations were deleterious, affecting protein function. Amino acid deletion is shown with green box. In this study, 11 different mutations in PmrA (S29G, T31S, T79A, T85A, L116V, R118F, E126K, I128N, R139P, G144S, and T151A) were observed in 24 *mcr*-negative colistin-resistant isolates. Of them, four mutations, namely, T79A, R118F, R139P, and T151A, were deleterious, affecting protein function. In addition, we found similar mutations with previous study in PmrA (S29G, T31S, I128N, and G144S) (Luo et al., 2017). However, we also found many novel mutations in PmrA (T79A, T85A, L116V, R118F, E126K, R139P, and T151A). The seven mutations have not been reported in *mcr*-negative colistin-resistant *E. coli*. These mutations may confer colistin resistance in *mcr*-negative *E. coli*.

The *pmrB* gene encodes a short 363–amino acid transmembrane protein. Four mutations T235N (one *mcr*-negative isolate), D283G (11 *mcr*-negative isolates), V351I (one *mcr*-negative isolate), and Y358N (10 *mcr*-negative isolates) were detected. We found similar mutations in PmrB (D283G, V351I, and Y358N) with previous studies (Delannoy et al., 2017; Luo et al., 2017).

The *phoP* gene encodes a short 456–amino acid transmembrane protein. One mutation A416T (one *mcr-1*–negative isolate) was detected. The *phoQ* gene encodes a short 486–amino acid transmembrane protein. One mutation K46T (one *mcr*-negative isolate) was detected. The mutation K46T [PROVEAN score = -3.746 (cutoff = -2.5)] was deleterious, affecting protein function.

### Domain Architectures of *PmrAB*, *PhoPQ*, and *MgrB* in *mcr*-Negative Isolates

**FIGURE 6:**
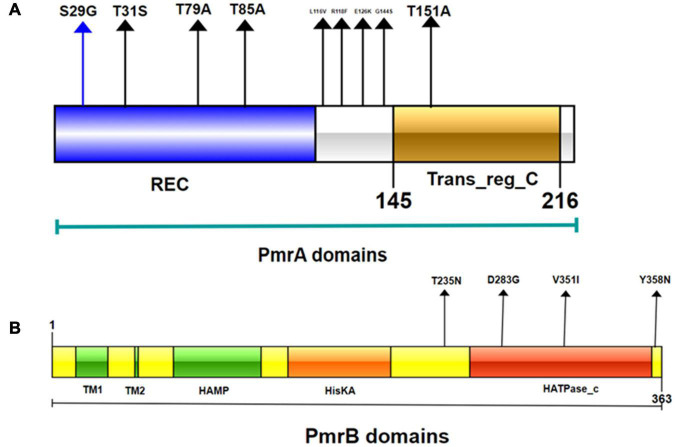
Domains of the PmrA/PmrB two-component system and positions of all mutations conferring colistin resistance to *E. coli.* **(A)** PmrA domains, cheY-homologous receiver domain [REC]; aa 1-112. Transcriptional regulatory protein, C-terminal domain [Trans_reg_C]; aa 145–216. S29G, T31S, T79A, T85A, L116V, R118F, E126K, I128N, G144S, and T151A represent mutation site. **(B)** PmrB domains, first transmembrane domain [TM1]; aa 15–34; second transmembrane domain [TM2]; aa 66–68. histidine kinases, adenylyl cyclases, methyl binding proteins, phosphatases [HAMP domain]; aa 89-141. Histidine kinase A (phosphoacceptor) domain [HisKA]; aa 142–202. Histidine kinase-like ATPases [HATPase_c]; aa 249-357. D283G, V351I, and Y358N represent smutation site.

SMART analysis was performed to determine the domain architectures of PmrA, PmrB, PhoP, PhoQ, and MgrB. As shown in Figure 6, we found that mutations have occurred in different domains of both PmrA and PmrB. In addition, *pmrA* appears to be the most commonly mutated gene in *E. coli.* The mutations T79A, R118F, R139P, and T151A were unique in PmrA. T91A is located in the cheY-homologous receiver domain. This domain contains a phosphoacceptor site that is phosphorylated by histidine kinase homologs. R118F, R139P, and T151A were located in transcriptional regulatory protein, C terminal. This domain is almost always found associated with the response regulator receiver domain. It may play a role in DNA binding. SMART analysis suggests that mutations T235N, V351I, and Y358N occurred in the HATPase_c domain in PmrB. The mutation V8A located in transmembrane region in MgrB, which starts at position 7 and ends at position 24. In summary, this study revealed diverse genetic mutations in two-component systems PmrAB and PhoPQ and their regulators MgrB in *mcr*-negative colistin-resistant *E. coli* isolates from Sichuan, China. However, mutated PmrB proteins do not contribute to colistin resistance (Wang et al., 2021). Although PmrAB, MgrB, and PhoPQ may be responsible for *mcr*-negative colistin-resistant *E. coli*, the mechanisms of colistin resistance appear to be highly diverse. These results suggest that the mechanisms underlying colistin resistance remain to be discovered in *E. coli*.

## Conclusion

The finding of this study showed the high prevalence of colistin in farms in Sichuan, China. The conjugation experiment and plasmid replicon type analysis suggest that the *mcr-1* gene is more likely to be horizontally transferred. The ST diversity of the *mcr-1* indicated a scattered and non-clonal. In addition, this study demonstrates diverse genetic mutations in two-component systems PmrAB and PhoPQ and their regulators MgrB of *mcr*-negative colistin-resistant *E. coli* isolates. In this study, we found several novel mutations, which have not been reported in *mcr*-negative colistin-resistant *E. coli*. These substitutions may confer colistin resistance in *mcr*-negative *E. coli*. To confirm our findings, further studies elucidating the resistance mechanism of *mcr*-negative *E. coli* to colistin are under way.

## Data Availability Statement

The raw data supporting the conclusions of this article will be made available by the authors, without undue reservation.

## Author Contributions

FL conceived and designed the experiments and wrote the manuscript. PC, XL, RL, and HL provided assistance of the experiments and collected the samples. XZ supervised the experiments and revised the manuscript. All authors contributed to the article and approved the submitted version.

## Conflict of Interest

The authors declare that the research was conducted in the absence of any commercial or financial relationships that could be construed as a potential conflict of interest.

## Publisher’s Note

All claims expressed in this article are solely those of the authors and do not necessarily represent those of their affiliated organizations, or those of the publisher, the editors and the reviewers. Any product that may be evaluated in this article, or claim that may be made by its manufacturer, is not guaranteed or endorsed by the publisher.

## Footnotes

1
https://clsi.org/

2
http://sift.jcvi.org

3
http://smart.embl.de/
