# Supplementary material for: Molecular Epidemiology and Colistin-Resistant Mechanism of mcr-Positive and mcr-Negative Escherichia coli Isolated From Animal in Sichuan Province, China
Source: Front Microbiol. 2022 Mar 29;13:818548. doi: 10.3389/fmicb.2022.818548 (PMC9002323; doi:10.3389/fmicb.2022.818548)
Supplement: Supplementary file 1 [file Data_Sheet_1.PDF]

Supplementary Data

Table S1. The primers of *16S rRNA* and *mcr* gene used in this study

Primer name	Sequence	Fragment Size (bp)
mcr-1-F	AGTCCGTTTGTTCTTGTGGC	320
mcr-1-R	AGATCCTTGGTCTCGGCTTG	
mcr-2-F	CAAGTGTGTTGGTCGCAGTT	715
mcr-2-R	TCTAGCCCGACAAGCATACC	
mcr-3-F	AAATAAAAATTGTTCCGCTTATG	929
mcr-3-R	AATGGAGATCCCCGTTTTT	
mcr-4-F	TCACTTTCATCACTGCGTTG	1115
mcr-4-R	TTGGTCCATGACTACCAATG	
mcr-5-F	ATGCGGTTGTCTGCATTTATC	1644
mcr-5-R	TCATTGTGGTTGTCCTTTTCTG	
mcr-6-F	AGCTATGTCAATCCCGTGAT	252
mcr-6-R	ATTGGCTAGGTTGTCAATC	
mcr-7-F	GCCCTTCTTTTCGTTGTT	551
mcr-7-R	GGTTGGTCTCTTTCTCGT	
mcr-8-F	TCAACAATTCTACAAAGCGTG	856
mcr-8-R	AATGCTGCGCGAATGAAG	
mcr-9-F	TTCCCTTTGTTCTGGTTG	1011
mcr-9-R	GCAGGTAATAAGTCGGTC	
16S rRNA-F	AGAGTTTGATCATGGCTCAG	1530
16S rRNA-R	TACGGTTACCTTGTTACGACTT	

Table S2. The primers of resistant gene used in this study

Primer name	Sequence	Fragment Size (bp)
bla$_{CTX-M}$-F	TTTGCGATGTGCAGTACCAGTAA	556
bla$_{CTX-M}$-R	CGATATCGTTGGTGGTGCCATA	
bla$_{TEM}$-F	ATTCTTGAAGACGAAAGGGC	530
bla$_{TEM}$ -R	ACGCTCAGTGGAACGAAAAC	
bla$_{SHV}$-F	CACTCAAGGATGTATTGTG	870
bla$_{SHV}$-R	TTAGCGTTGCCAGTGCTCG	
strA-F	TGCTCCTCTTCTCCATCC	197
strA-R	CGCCGCCAATATGTTCTA	
strB-F	ATGATGCAGATCGCCATGTA	326
strB-R	CGGTCGTAGAGGCAATCTG	
aacC2-F	ACTGTGATGGGATACGCGTC	237
aacC2-R	CTCCGTCAGCGTTTCAGCTA	
aacC4 -F	CTTCAGGATGGCAAGTTGGT	286
aacC4 -R	TCATCTCGTTCTCCGCTCAT	
tetA-F	GTAATTCTGAGCACTGTCGC	937
tetA-R	CTGCCTGGACAACATTGCTT	
tetB-F	CTCAGTATTCCAAGCCTTTG	416
tetB-R	CTAAGCACTTGTCTCCTGTT	
tetC-F	TCTAACAATGCGCTCATCGT	570
tetC-R	GGTTGAAGGCTCTCAAGGGC	
qnrS-F	GCAAGTTCATTGAACAGGGT	428
qnrS-R	TCTAAACCGTCGAGTTCGGCG	
oqxAB-F	CCGCACCGATAAATTAGTCC	313
oqxAB-R	GGCGAGGTTTTGATAGTGGA	
qepA-F	GCAGGTCCAGCAGCGGGTAG	218
qepA-R	CTTCCTGCCCGAGTATCGTG	
aac(6')-Ib-cr-F	GGCGAGGTTTTGATAGTGGA	260
aac(6')-Ib-cr-R	TTGGAAGCGGGGACGGAM	
floR-F	CACGTTGAGCCTCTATAT	856
floR-R	ATGCAGAAGTAGAACGCG	

Primer name	Sequence	Fragment Size (bp)
sul1-F	ATCTCACCGACCACTCCTTC	576
sul1-R	ACGCCGACTTCAGCTTTT	
sul2-F	CATCATTTTCGGCATCGTC	793
sul2-R	TCTTGCGGTTTCTTTCAGC	
sul3-F	AGATGTGATTGATTTGGGAGC	443
sul3-R	TAGTTGTTTCTGGATTAGAGCCT	

Table S3. The primer of *pmrAB*, *mgrB*, and *phoPQ* gene used in this study

Primer name	Sequence	Fragment Size (bp)
pmrA-F	AGTTTTCCTCATTCGCGACCA	714
pmrA-R	TACCAGGCTGCGGATGATATTCT	
pmrB-F	GGATGGCCTGATGTGACGCTGTC	1312
pmrB-R	GCGCGGCTTTGGCTATA	
mgrB-F	CACGAATATCGACATAGTTAG	275
mgrB-R	TATTCTACCACTGCTGGAGAG	
phoP-F	GCCAGTACCGCCAGCTTAA	1798
phoP-R	CTCGCCACGTAACAGCCGAA	
phoQ-F	GGCACAATATCCCCAAGAAGT	1595
PhoQ-R	ATCCACAGGCTGGTATCTGCA	

Table S4. The primers of housekeeping gene used in this study

Primer name	Sequence	Fragment Size (bp)
adk-F	AGTTTTCCTCATTCGCGACCA	583
adk-R	TACCAGGCTGCGGATGATATTCT	
fumc-F	GGATGGCCTGATGTGACGCTGTC	806
fumc-R	GCGCGGCTTTGGCTATA	
gyrB-F	GCCAGTACCGCCAGCTTAA	911
gyrB-R	CTCGCCACGTAACAGCCGAA	
icd-F	GGCACAATATCCCCAAGAAGT	878
icd-R	ATCCACAGGCTGGTATCTGCA	
mdh-F	CACGAATATCGACATAGTTAG	932
mdh-R	TATTCTACCACTGCTGGAGAG	
purA-F	TCGGTAACGGTGTTGTGCTG	816
purA-R	CATACGGTAAGCCACGCAGA	
recA-F	ACCTTTGTAGCTGTACCACG	780
recA-R	AGCGTGAAGGTAAAACCTGTG	

Table S5. Colistin MIC of colistin-resistant *E. coli* in this study

Strains	Origin	Colistin MIC (μg/ml)	Strains	Origin	Colistin MIC (μg/ml)
SC5	Chicken	4	SC35	Chicken	8
SC7	Chicken	4	SC36	Chicken	4
SC8	Chicken	4	SC42	Pig	4
SC10	Chicken	8	SC51	Pig	4
SC11	Chicken	4	SC53	Pig	4
SC24	Chicken	4	SC55	Pig	4
SC25	Chicken	4	SC57	Pig	4
SC27	Chicken	4	SC58	Pig	4
SC28	Chicken	4	SC60	Pig	4
SC31	Chicken	4	SC64	Pig	4
SC32	Chicken	8	SC66	Pig	4
SC33	Chicken	4	SC67	Pig	4
SC34	Chicken	4	SC68	Pig	4

Strains	Origin	Colistin MIC (µg/ml)	Strains	Origin	Colistin MIC (µg/ml)
SC69	Pig	4	SC167	Dog	4
SC70	Pig	4	SC168	Dog	4
SC71	Pig	4	SC169	Dog	4
SC72	Pig	4	SC172	Dog	4
SC76	Pig	4	SC173	Dog	4
SC77	Pig	4	SC175	Dog	4
SC78	Pig	4	SC177	Dog	4
SC81	Pig	128	SC185	Dog	4
SC85	Pig	4	SC186	Dog	4
SC86	Pig	4	SC187	Cattle	4
SC105	Pig	4	SC189	Cattle	4
SC111	Pig	4	SC190	Cattle	4
SC115	Pig	4	SC191	Cattle	4
SC120	Pig	4	SC193	Cattle	4
SC121	Pig	4	SC195	Cattle	8
SC124	Pig	4	SC197	Cattle	4
SC127	Pig	4	SC200	Cattle	4
SC131	Dog	8	SC201	Cattle	4
SC136	Dog	64	SC205	Cattle	4
SC140	Dog	4	SC207	Cattle	4
SC141	Dog	4	SC208	Cattle	4
SC147	Dog	8	SC209	Cattle	4
SC151	Dog	4	SC211	Cattle	4
SC157	Dog	4	SC213	Cattle	4
SC158	Dog	4	SC214	Cattle	4
SC160	Dog	4	SC215	Cattle	8
SC161	Dog	4	SC216	Cattle	8
SC163	Dog	4	SC217	Cattle	8
SC164	Dog	4	SC218	Cattle	4
SC165	Dog	4	SC219	Cattle	4
SC166	Dog	4	SC221	Cattle	4

Strains	Origin	Colistin MIC (μg/ml)	Strains	Origin	Colistin MIC (μg/ml)
SC223	Cattle	4	SC232	Cattle	4
SC225	Cattle	4	SC233	Cattle	4
SC227	Cattle	4	SC234	Cattle	4
SC228	Cattle	4	SC245	Cattle	4
SC229	Cattle	4	SC250	Cattle	4
SC230	Cattle	4	SC251	Cattle	4
SC231	Cattle	8			

Table S6 The resistance phenotype of *mcr-1*-positive *E. coli.*

Strains	Origin	Multiple antibiotic resistance	Resistance phenotype
SC5	Chicken	11	AMC-FEP-CAZ-AMP-SAM-FOX-S-CRO-CXM-SF-TE
SC7	Chicken	9	C-AMP-SAM-FOX-S-CRO-CXM-SF-TE
SC10	Chicken	14	K-C-ATM-AMP-SAM-FOX-DO-S-CRO-FFC-CXM-SF-CN-TE
SC11	Chicken	13	K-C-ATM-AMP-SAM-FOX-DO-S-CRO-FFC-CXM-CN-TE
SC24	Chicken	18	CIP-LEV-K-FEP-CAZ-C-ATM-AMP-SAM-FOX-DO-S-CRO-FFC-CXM-SF-CN-TE
SC25	Chicken	14	CIP-LEV-K-FEP-C-ATM-AMP-FOX-DO-CRO-FFC-CXM-SF-TE
SC27	Chicken	14	CIP-LEV-K-FEP-C-ATM-AMP-FOX-DO-CRO-FFC-CXM-SF-TE
SC31	Chicken	0	-
SC33	Chicken	0	-
SC34	Chicken	8	CIP-LEV-FOS-AMP-S-CRO-CXM-TE
SC35	Chicken	12	CIP-LEV-K-C-AMP-S-CRO-FFC-CXM-SF-CN-TE
SC36	Chicken	0	-
SC51	Pig	7	AMP-DO-S-FFC-SF-CN-TE
SC57	Pig	6	FOS-AMP-CRO-CXM-SF-TE
SC60	Pig	3	DO-SF-TE

Strains	Origin	Multiple antibiotic resistance	Resistance phenotype
SC64	Pig	6	C-AMP-S-FFC-SF-TE
SC66	Pig	4	AMP-DO-SF-TE
SC67	Pig	10	K-FEP-C-ATM-AMP-CRO-FFC-CXM-SF-TE
SC68	Pig	9	K-C-AMP-S-CRO-FFC-CXM-SF-TE
SC71	Pig	4	C-DO-SF-TE
SC72	Pig	3	DO-SF-TE
SC78	Pig	2	S-SF
SC85	Pig	4	AMC-AMP-S-SF
SC86	Pig	3	DO-SF-TE
SC111	Pig	7	C-AMP-DO-S-FFC-SF-TE
SC121	Pig	7	K-C-DO-S-FFC-SF-TE
SC124	Pig	8	K-C-AMP-DO-S-FFC-SF-TE
SC127	Pig	3	DO-SF-TE
SC131	Dog	9	C-AMP-DO-CRO-FFC-CXM-SF-CN-TE
SC136	Dog	7	C-DO-S-CRO-SF-CN-TE
SC140	Dog	1	S
SC141	Dog	0	-
SC147	Dog	4	FOX-S-SF-TE
SC151	Dog	1	S
SC157	Dog	2	ATM-SF
SC158	Dog	5	C-AMP-DO-SF-TE
SC160	Dog	0	-
SC163	Dog	3	S-SF-CN
SC164	Dog	5	C-AMP-DO-S-TE
SC172	Dog	6	AMP-S-CRO-FFC-CXM-SF
SC173	Dog	1	SF
SC175	Dog	1	SF
SC207	Cattle	4	C-AMP-FFC-TE
SC216	Cattle	6	C-AMP-DO-FFC-SF-TE
SC217	Cattle	5	CIP-AMP-S-SF-CN

Strains	Origin	Multiple antibiotic resistance	Resistance phenotype
SC218	Cattle	6	K-AMP-DO-S-SF-TE
SC221	Cattle	5	C-AMP-DO-FFC-TE
SC223	Cattle	4	C-AMP-FFC-SF
SC225	Cattle	5	K-C-AMP-FFC-SF
SC227	Cattle	4	AMP-DO-SF-TE
SC229	Cattle	4	C-S-FFC-TE
SC230	Cattle	3	C-S-SF
SC231	Cattle	6	K-C-AMP-S-FFC-SF
SC234	Cattle	9	CIP-LEV-C-AMP-S-FFC-SF-CN-TE

Abbreviations: AMP, ampicillin; CXM, cefuroxime; CAZ, ceftazidime; FEP, cefepime; CRO, ceftriaxone; FOX, cefoxitin; ATM, aztreonam; SAM, ampicillin–sulbactam; AMC, amoxicillin–clavulanic acid; CN, gentamicin; AK, amikacin; K, kanamycin; STR, streptomycin; CIP, ciprofloxacin; LEV, levofloxacin; TE, tetracycline; DO, doxycycline; TGC, tigecycline; C, chloramphenicol; FFC, florfenicol; FOS, fosfomycin; SF, sulfisoxazole; F, nitrofurantoin.
